# Enzymatically-crosslinked gelatin hydrogels containing paenipeptin and clarithromycin against carbapenem-resistant pathogen in murine skin wound infection

**DOI:** 10.1186/s12866-021-02383-z

**Published:** 2021-11-24

**Authors:** Sun Hee Moon, Yihong Kaufmann, Ryoichi Fujiwara, En Huang

**Affiliations:** 1 Department of Environmental and Occupational Health, University of Arkansas for Medical Sciences, 4301 West Markham Street, Little Rock, AR 72205 USA; 2 Department of Pharmaceutical Sciences, University of Arkansas for Medical Sciences, 4301 West Markham Street, Little Rock, AR 72205 USA

**Keywords:** Carbapenem-resistance, Wound infection, Paenipeptin, Clarithromycin, Hydrogel

## Abstract

**Background:**

The recent rise and spread of carbapenem-resistant pathogens pose an urgent threat to public health and has fueled the search for new therapies. Localized delivery of topical antibiotics is an alternative for the treatment of infected wounds caused by drug-resistant pathogens. In this study, we aimed to develop antimicrobial-loaded hydrogels for topical treatment of wound infections in a murine skin wound infection.

**Results:**

Paenipeptin analogue 1, a linear lipopeptide, potentiated clarithromycin against multidrug-resistant *Acinetobacter baumannii*, *Enterobacter cloacae*, *Escherichia coli*, and *Klebsiella pneumoniae*. Enzymatically-crosslinked gelatin hydrogels were developed to encapsulate paenipeptin analogue 1 and clarithromycin. The encapsulated antimicrobials were gradually released from hydrogels during incubation, reaching 75.43 and 53.66% for paenipeptin and clarithromycin, respectively, at 24 h. The antimicrobial-loaded hydrogels containing paenipeptin and clarithromycin synergistically resulted in 5-log reduction in carbapenem-resistant *A. baumannii* within 6 h in vitro. Moreover, the antimicrobial-loaded hydrogels reduced 3.6- and 2.5-log of carbapenem-resistant *A. baumannii* when treated at 4 or 20 h post infection, respectively, in a murine skin wound infection.

**Conclusions:**

Enzymatically-crosslinked gelatin hydrogels loaded with paenipeptin analogue 1 and clarithromycin exhibited potent therapeutic efficacy against carbapenem-resistant *A. baumannii* in murine skin wound infection.

**Supplementary Information:**

The online version contains supplementary material available at 10.1186/s12866-021-02383-z.

## Background

Bacterial infections caused by carbapenem-resistant pathogens are difficult to treat and have been recognized as an urgent threat [1, 2]. It was estimated that carbapenem-resistant *Acinetobacter* caused 8500 cases of infections and 700 deaths in the United States in 2017 [3]. Drug-resistant *Acinetobacter* is a challenging threat to patients in healthcare facilities. *Acinetobacter* can cause lung, wound, urinary tract, and bloodstream infections [3]. Wound infections caused by multidrug-resistant pathogens, including *Acinetobacter baumannii*, are serious problems with regard to morbidity and mortality [4–6].

Localized delivery of topical antibiotics is an alternative to systemic antibiotics for the treatment of infected wounds [7]. The use of topical medications allows for the delivery of high concentrations of antimicrobials at the site of infection. Antimicrobial-loaded hydrogels can serve as topical antimicrobial carriers for the treatment of wound infections [8, 9]. For example, the lipopeptide antibiotic colistin was encapsulated in glycol chitosan-based hydrogels for the treatment of infection in mice. The localized release of colistin from hydrogels exhibited potent activity against *Pseudomonas aeruginosa* in the in vivo animal “burn” infection model [8]. Similarly, topically delivered moxifloxacin reduced *P. aeruginosa* and *Staphylococcus aureus* wound infections and prompted wound healing [9].

Gelatin is a mixture of water-soluble proteins obtained by collagen hydrolysis. Gelatin has great biocompatibility and biodegradability and has been extensively used as a biomaterial for tissue engineering and drug delivery [10, 11]. Gelatin hydrogels can be made using chemical and enzymatic crosslinking to increase mechanical and thermal strength. However, the chemical crosslinking agents, such as glutaraldehyde, may be potentially cytotoxic [12], which limits its biomedical applications. Biochemical crosslinking approaches are more biocompatible when compared to chemical crosslinking. Microbial transglutaminase has been used to enzymatically form thermally stable gelatin hydrogels to be used as a tissue engineering scaffold [12] and a biomimetic tissue sealant [13, 14]. The favorable biocompatibility of transglutaminase crosslinked gelatin hydrogels makes it a promising drug carrier for the delivery of topical antimicrobial agents.

Paenipeptins are synthetic linear lipopeptides that sensitize Gram-negative pathogens to antibiotics that have little effect when used alone [15]. We previously reported that paenipeptin analogues potentiated clarithromycin and rifampicin against 10 carbapenem-resistant pathogens, including five isolates of *A. baumannii* and five isolates of *Klebsiella pneumoniae* [16]. Moreover, systemic administration of paenipeptin analogues in combination with clarithromycin or rifampicin was effective against *mcr-1*-mediated polymyxin-resistant *Escherichia coli* in a neutropenic murine thigh infection model [17]. In this study, we aimed to develop antimicrobial-loaded hydrogels for localized delivery of paenipeptin-clarithromycin mixture for topical treatment of wound infections in a murine skin wound infection.

## Results and discussion

### Paenipeptin analogue 1 potentiated clarithromycin against multidrug-resistant pathogens

The MIC range, MIC_50_, and MIC_90_ of paenipeptin analogue 1, clarithromycin, and their combination are summarized in Table 1. Paenipeptin at sub-inhibitory concentration (4 μg/ml) enhanced the activity of clarithromycin against *A. baumannii*, *Enterobacter cloacae*, *E. coli*, and *K. pneumoniae*. The MIC_90_ of clarithromycin when used alone was > 32 μg/ml for all tested isolates in these four genera, whereas the MIC_90_ of clarithromycin in the presence of paenipeptin analogue 1 (4 μg/ml) ranged from 0.0625 to 8 μg/ml. Paenipeptin promoted the uptake of clarithromycin by disrupting the outer membrane of the susceptible Gram-negative pathogens [17]. However, paenipeptin failed to increase the activity of clarithromycin against *P. aeruginosa*. The results were consistent with the previous reports. For example, NAB741, a polymyxin B derivative, potentiated clarithromycin and other antibiotics against *E. coli*, *K. pneumoniae* and *A. baumannii* [18], but not *P. aeruginosa* [19]. Similarly, a linear cationic peptide, unacylated tridecaptin A1 (H-TriA1), substantially lowered the MIC of rifampicin against *E. coli* and *K. pneumoniae* strains but not *P. aeruginosa* [20]. However, the mechanism of lacking synergistic effect against *P. aeruginosa* remains unknown.

**Table 1:** Minimum inhibitory concentration (MIC) of clarithromycin (CLR) with or without paenipeptin analogue 1 (Paen1) against 70 isolates in the Gram-Negative Carbapenemase Detection panel from the FDA-CDC Antibiotic-Resistance (AR) Bank

Species	No. of Isolates	MIC of Paen1 (μg/ml)			MIC of CLR (μg/ml)			MIC of CLR (μg/ml) (in the presence of Paen1 at 4 μg/ml)		
		Range	50%	90%	Range	50%	90%	Range	50%	90%
*A. baumannii*	14	2− > 32	32	> 32	4− > 32	32	> 32	0.0019 − 8	0.03125	8
*E. cloacae*	9	4− > 32	16	> 32	> 32	> 32	> 32	0.0019 − 0.25	0.03125	0.25
*E. coli*	13	4 − 32	16	32	> 32	> 32	> 32	0.0019 − 0.0625	0.03125	0.0625
*K. pneumoniae*	22	32− > 32	> 32	> 32	> 32	> 32	> 32	0.03125 − 8	0.25	8
*P. aeruginosa*	12	8− > 32	32	32	> 32	> 32	> 32	16− > 32	> 32	> 32

### Release kinetics and time-kill kinetics of antimicrobial-loaded gelatin hydrogels in vitro

**Fig. 1:**
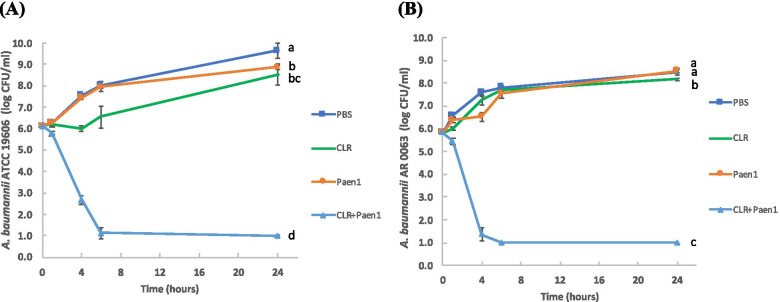
Time-kill kinetics of **A ***Acinetobacter baumannii* ATCC 19606 and **B** carbapenem-resistant *A. baumannii* AR 0063 after exposure to antimicrobial-loaded hydrogels in tryptic soy broth. PBS: hydrogel in PBS without antimicrobials (negative control); CLR: hydrogel with 0.1 mg/ml clarithromycin; Paen1: hydrogel with 0.1 mg/ml paenipeptin analogue 1; CLR + Paen1: hydrogel with 0.1 mg/ml clarithromycin and 0.1 mg/ml paenipeptin analogue 1. Values are expressed as means (4 independent repeats), and error bars represent standard deviations. Means with different letters are significantly different between groups (*p* < 0.05) at the final data points at 24 h

The encapsulated antimicrobials were gradually released from hydrogels during incubation, reaching 75.43 and 53.66% for paenipeptin and clarithromycin, respectively, at 24 h (Table 2). In the in vitro time-kill kinetics tests in tryptic soy broth, single treatments by hydrogels loaded with paenipeptin analogue 1 or clarithromycin reduced the growth of *A. baumannii* ATCC 19606 when compared with the PBS control, but the final populations of the bacterium exceeded the inoculum level and reached 8.5 log CFU/ml at 24 h. Similarly, paenipeptin analogue 1 slowed the growth of *A. baumannii* AR 0063 by 6 h, but the final population of the bacterium reached a similar level as the PBS control at 24 h. Clarithromycin slightly reduced the growth of *A. baumannii* AR 0063 but the final population of the bacterium also exceeded the inoculum level and reached 8.0 log CFU/ml at 24 h. In contrast, the combined treatments with both compounds synergistically resulted in 5-log reduction in both strains within 6 h (Fig. 1).

**Table 2:** HPLC quantification of antimicrobials released from gelatin hydrogel containing paenipeptin and clarithromycin

Time (h)	Paenipeptin analogue 1		Clarithromycin	
	μg	Release percentage (%)	μg	Release percentage (%)
3	21.85 ± 2.58	24.28	17.00 ± 0.42	18.89
4	27.91 ± 2.45	31.00	19.34 ± 0.43	21.49
6	38.09 ± 1.87	42.33	26.51 ± 1.04	29.45
8	42.51 ± 1.68	47.24	29.61 ± 2.07	32.90
10	50.35 ± 0.60	55.95	35.02 ± 1.50	38.91
24	67.89 ± 7.47	75.43	48.29 ± 5.84	53.66

### In vivo therapeutic efficacy of antimicrobial-loaded gelatin hydrogels

**Fig. 2:**
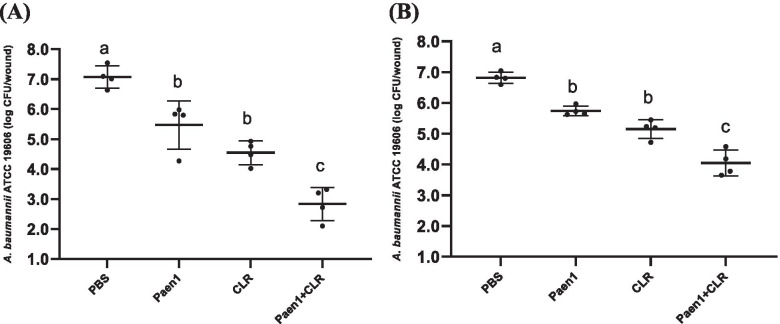
Efficacy of gelatin hydrogels containing paenipeptin analogue 1, clarithromycin, or their combination against *Acinetobacter baumannii* ATCC 19606 in murine skin wound infection (*n* = 4, female CD-1 mice). **A** Treatments at 4 h post infection; **B** Treatments at 20 h post infection. PBS: hydrogel in PBS without antimicrobials (negative control); Paen1: hydrogel with 0.1 mg/ml paenipeptin analogue 1; CLR: hydrogel with 0.1 mg/ml clarithromycin; Paen1+ CLR: hydrogel with 0.1 mg/ml paenipeptin analogue 1 and 0.1 mg/ml clarithromycin. Means with different letters are significantly different between groups (*p* < 0.05)

In the murine skin infection model, single treatments started at 4 h post infection by gelatin hydrogels containing paenipeptin and clarithromycin resulted in 1.6- and 2.5-log reduction of *A. baumannii* ATCC 19606, respectively. The combined treatment started at 4 h post infection led to 4.2 log-reduction whereas the combined treatment started at 20 h post infection resulted in 2.8-log reduction (Fig. 2). The results indicated that bacterial pathogens in the established wounds (20 h post infection) became less susceptible to antimicrobial treatments than that in the freshly infected wounds (4 h post infection). Therefore, the initiating time for treatment after infection affected the therapeutic efficacy. It was reported that the common topical antimicrobial agents, including mupirocin and bacitracin, had reduced efficacy against methicillin-resistant *S. aureus* (MRSA) when treatment was initialed at 24 h post infection compared to 4 h after infection in a superficial murine wound model [21].

**Fig. 3:**
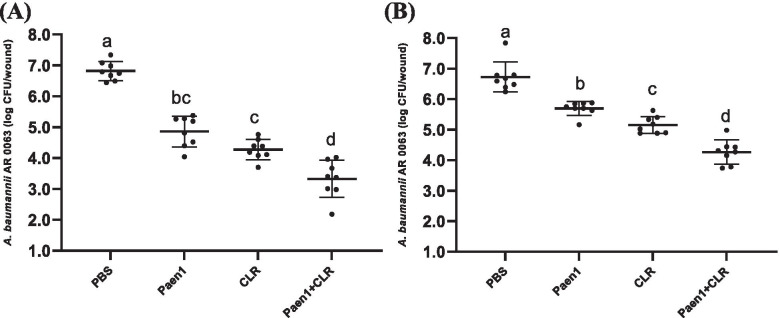
Efficacy of gelatin hydrogels containing paenipeptin analogue 1, clarithromycin, or their combination against carbapenem-resistant *Acinetobacter baumannii* AR0063 in murine skin wound infection (*n* = 8, equal number of female and male CD-1 mice). **A** Treatments at 4 h post infection; **B** Treatments at 20 h post infection. PBS: hydrogel in PBS without antimicrobials (negative control); Paen1: hydrogel with 0.1 mg/ml paenipeptin analogue 1; CLR: hydrogel with 0.1 mg/ml clarithromycin; Paen1+ CLR: hydrogel with 0.1 mg/ml paenipeptin analogue 1 and 0.1 mg/ml clarithromycin. Means with different letters are significantly different between groups (*p* < 0.05)

We further studied the therapeutic efficacy against a carbapenem-resistant strain, *A. baumannii* AR0063. Similarly, when the treatments started at 4 h post infection, single treatments by hydrogels with paenipeptin and clarithromycin resulted in 2.0- and 2.6-log reduction of *A. baumannii* AR0063, respectively. The combined treatment started at 4 h post infection led to 3.6-log reduction whereas the combined treatment started at 20 h post infection resulted 2.5-log reduction (Fig. 3). Other antimicrobial peptides have been studied as topical antimicrobial agents against *A. baumannii* infections. For example, an engineered peptide derived from a bacteriophage lysin reduced the bacterial burden of multidrug-resistant *A. baumannii* by 2 log in 2 h when the treatment was initiated at 16 h after infection in a murine skin infection model [22].

### Skin irritation tests of antimicrobial-loaded gelatin hydrogels

Gelatin hydrogels containing both paenipeptin and clarithromycin at 0.1-0.4 mg/ml did not cause any signs of irritation after placing the hydrogels on the mice skin for 24 h (Table 3). However, at the end of 48 h extended incubation with a new hydrogel at 24 h, skin rash was observed in four out of six mice treated with hydrogels (Fig. S1) containing both paenipeptin and clarithromycin at the highest tested concentration (0.4 mg/ml), which was four times of the effective therapeutic concentration tested in the wound infection model.

**Table 3:** Skin irritation tests of gelatin hydrogels containing paenipeptin analogue 1 and clarithromycin on CD-1 mice

Concentration^a^ (mg/ml)	Number of mice with skin redness or rash after the treatment	
	24 h	48 h
0	0/6	0/6
0.1	0/6	0/6
0.2	0/6	0/6
0.4	0/6	4/6

^a^Paenipeptin analogue 1 and clarithromycin were tested at a final concentration of 0, 0.1, 0.2 or 0.4 mg/ml for each compound in the hydrogels

## Conclusions

Paenipeptin analogue 1 potentiated clarithromycin against multidrug-resistant Gram-negative pathogens, including *A. baumannii*, *E. cloacae*, *E. coli*, and *K. pneumoniae* but not *P. aeruginosa*. Moreover, we developed enzymatically-crosslinked gelatin hydrogels loaded with paenipeptin analogue 1 and clarithromycin. Gelation without using transglutaminase resulted in hydrogels that were not heat stable at 37 °C [12]. The enzymatic gelation method increased the thermal stability of hydrogels without using toxic crosslinking agents, such as glutaraldehyde. To the best of our knowledge, this is the first report using transglutaminase crosslinked gelatin hydrogels as a carrier for the delivery of topical antimicrobial agents. The antimicrobial-loaded hydrogels gradually released antibiotics and exhibited potent therapeutic efficacy against carbapenem-resistant *A. baumannii* in murine skin wound infection.

## Methods

### Synthesis of paenipeptin

Paenipeptin analogue 1 (> 95% purity) is a synthetic lipopeptide, which was custom synthesized by Genscript Inc. (Piscataway, NJ). The purity was determined by HPLC, and the peptide structure was verified by high resolution mass spectrometry (HRMS) and nuclear magnetic resonance (NMR) as described previously [15].

### Minimum inhibitory concentration determination

The minimum inhibitory concentration (MIC) of paenipeptin analogue 1, and clarithromycin (Sigma, St. Louis, MO) with or without paenipeptin analogue 1 was determined as described previously [15]. In this current study, we expanded the susceptibility tests against 70 isolates in the Gram-Negative Carbapenemase Detection panel obtained from the FDA-CDC Antibiotic-Resistance (AR) Bank. The majority of these isolates in the panel are resistant to carbapenem antibiotics [23].

### Preparation of antimicrobial-loaded enzymatically-crosslinked gelatin hydrogels

Enzymatically-crosslinked gelatin hydrogels were developed to encapsulate antimicrobials. The hydrogels contained paenipeptin analogue 1 (0.1 mg/ml), clarithromycin (0.1 mg/ml) or both compounds (each 0.1 mg/ml), 4% (w/v) gelatin (type A, 300 Bloom, Sigma), and transglutaminase (20 units/gram gelatin, Ajinomoto) in PBS (pH 7.2). The mixture was incubated at 37 °C for 1 h for gelation. Antimicrobial-loaded hydrogels were stored at 4 °C before use.

### Release kinetics of antimicrobials from hydrogels

To determine the release kinetics of antimicrobials, antimicrobial-loaded gelatin hydrogels were incubated at 37 °C for 24 h. Paenipeptin analogue 1 and clarithromycin released from hydrogels in aqueous solution were collected at 3, 4, 6, 8, 10, and 24 h during incubation, followed by quantification using HPLC as described previously [15]. Briefly, the gelatin hydrogels (0.9 ml) contained paenipeptin analogue 1 and clarithromycin at an equal concentration of 0.1 mg/ml. Release experiments (three independent replicates) were carried out in vitro during incubation at 37 °C. Quantification was performed using Waters Acquity UPLC with Waters BEH C_18_ Column (130 Å, 1.7 μm, 2.1 mm × 50 mm).

### Time-kill kinetics in vitro

The activity of antimicrobial-loaded gelatin hydrogels containing paenipeptin analogue 1 (0.1 mg/ml), clarithromycin (0.1 mg/ml), or both compounds (0.1 mg/ml for each) was evaluated in vitro using time-kill kinetics assay. Briefly, antimicrobial-loaded hydrogels (180 μl) was placed in 2 ml tryptic soy broth, which was inoculated with *A. baumannii* ATCC 19606 or the carbapenem-resistant strain, *A. baumannii* AR 0063, followed by incubation at 37 °C with agitation at 80 rpm for 24 h. Bacterial population was determined at 0, 1, 2, 4, 6 and 24 h. Data were analyzed using one-way analysis of variance (ANOVA) (GraphPad Prism, version 9.0; GraphPad Software Inc., San Diego, CA) and considered significant at *p* < 0.05.

### Therapeutic efficacy in murine infection model

All Animal experiments have been approved by the Institutional Animal Care and Use Committee (IACUC) at University of Arkansas for Medical Sciences (approval number 3922). The study was carried out in compliance with the ARRIVE guidelines. All experiments were performed in accordance with relevant guidelines and regulations. Mice were purchased from Charles River Laboratories. After hair removal, a partial-thickness skin wound was generated under isoflurane anesthetization by repeated brief touching the back skin of CD-1 mouse using a rotary tool (Dremel 8050) with a sterile sanding attachment until skin tissues were red and glistening [21]. After disinfection with alcohol wipes, an area of ~ 1 cm^2^ was inoculated with *A. baumannii* ATCC 19606 or *A. baumannii* AR 0063 (5.5-6.0 log CFU in 5 μl saline). At 4 or 20 h post infection, an antimicrobial-loaded gelatin hydrogel (0.9 ml), which was loaded on a bandage, was applied and secured to the infected wound. After antimicrobial treatment overnight for 18 h, mice were euthanized and wound tissues were collected for bacterial enumeration. All animals were euthanized by CO_2_ followed by cervical dislocation. Data were analyzed using one-way analysis of variance (ANOVA) (GraphPad Prism, version 9.0; GraphPad Software Inc., San Diego, CA) and considered significant at *p* < 0.05.

### Skin irritation tests

To test the potential skin irritation on mice (female and male CD-1, *n* = 6), gelatin hydrogels containing paenipeptin analogue 1 and clarithromycin at various concentrations (0.1, 0.2, or 0.4 mg/ml for both compounds) were placed and secured on mice skin for 24 h. After 24 h, the hydrogel was removed and replaced by a new hydrogel at the same concentration for another 24 h. All animals were euthanized by CO_2_ followed by cervical dislocation. Signs of irritation such as redness or rash were observed at 24 and 48 h.

## Supplementary Information

**Additional file 1: Figure S1.** Representative pictures of skin irritation tests of gelatin hydrogels containing paenipeptin analogue 1 and clarithromycin (0, 0.1, 0.2 or 0.4 mg/ml for both compounds) using CD-1 mice. Pictures were taken at 48 h after 2 repeated treatments at every 24 h. Black inks were used to mark the skin to guide the placement of the hydrogels. Red arrow at 0.4 mg/ml indicated the rash on the skin.

## Data Availability

All data generated or analyzed during this study are included in this published article.
