# Supplementary material for: Enzymatically-crosslinked gelatin hydrogels containing paenipeptin and clarithromycin against carbapenem-resistant pathogen in murine skin wound infection
Source: BMC Microbiol. 2021 Nov 24;21:326. doi: 10.1186/s12866-021-02383-z (PMC8611911; doi:10.1186/s12866-021-02383-z)
Supplement: Supplementary file 1 — Additional file 1: Figure S1. Representative pictures of skin irritation tests of gelatin hydrogels containing paenipeptin analogue 1 and clarithromycin (0, 0.1, 0.2 or 0.4 mg/ml for both compounds) using CD-1 mice. Pictures were taken at 48 h after 2 repeated treatments at every 24 h. Black inks were used to mark the skin to guide the placement of the hydrogels. Red arrow at 0.4 mg/ml indicated the rash on the skin. [file 12866_2021_2383_MOESM1_ESM.docx]

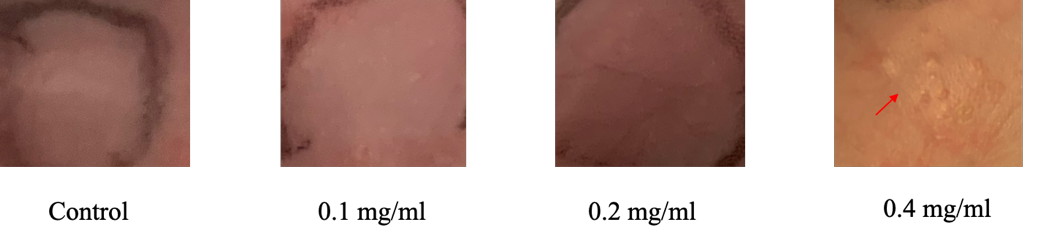


**Figure S1**. Representative pictures of skin irritation tests of gelatin hydrogels containing paenipeptin analogue 1 and clarithromycin (0, 0.1, 0.2 or 0.4 mg/ml for both compounds) using CD-1 mice. Pictures were taken at 48 h after 2 repeated treatments at every 24 h. Black inks were used to mark the skin to guide the placement of the hydrogels. Red arrow at 0.4 mg/ml indicated the rash on the skin.
